# Society News

**DOI:** 10.1093/nop/npag060

**Published:** 2026-08-18

**Forthcoming Meetings** 

**Congress of Neurological Surgeons Annual Meeting** 

October 31-November 4, 2026—Washington, DC, USA

https://www.cns.org/annualmeeting

**Society of Neuro-Oncology Annual Meeting and Education Day** 

November 12-15, 2026—Philadelphia, PA, USA

https://sno2026.oa-event.com/

**American Association for Cancer Research (AACR) Annual Meeting** 

April 2-7, 2027—Orlando, FL, USA

https://www.aacr.org/meeting/aacr-annual-meeting-2027/

**Annual Meeting of American Academy of Neurology** 

May 1-5, 2027—Washington, DC, USA

https://www.aan.com/events/upcoming-events

